# Putrescence to Quintessence: An Atypical Presentation of Multiple Osteoporotic Spinal Fractures Masquerading as Multiple Myeloma

**DOI:** 10.7759/cureus.20788

**Published:** 2021-12-29

**Authors:** Vivek A Ojha, Vibhu Bahl, Shobha C Ramachandra, Akila Prashant

**Affiliations:** 1 Department of Biochemistry, Employees’ State Insurance Corporation (ESIC) Medical College and Hospital, Patna, IND; 2 Department of Orthopedics, Indraprastha Apollo Hospital, Delhi, IND; 3 Department of Biochemistry, Jagadguru Sri Shivarathreeshwara (JSS) Medical College, Mysore, IND

**Keywords:** spinal fracture, crp, osteoporosis, multiple myeloma, beta 2 microglobulin

## Abstract

A 64-year-old male patient presented with multiple osteoporotic spinal fractures of unknown origin. He was provisionally diagnosed with multiple myeloma based on biochemical and radiological findings. The patient presented in a very frail condition with a questionable outcome but showed a remarkable recovery from being frail to relatively fit. His baseline characteristics including magnetic resonance imaging of the dorsolumbar spine, beta 2 microglobulins, and C-reactive protein improved. The diagnosis was later changed to multiple spinal osteoporotic fractures. In this case report, we highlight that, although it is a good practice to have a single working diagnosis, when the diagnosis is challenging, a holistic approach should be followed to prevent medical and diagnostic miscalculations.

## Introduction

Multiple myeloma (MM) presents with varied phenotypes, and multiple spinal osteoporotic fractures with constitutional symptoms suggest malignancy [1]. Apparent immunoglobulin (Ig)G, IgA, IgM, and IgE aberrations in serum electrophoresis, along with the presence of light and heavy chains, suggest the diagnosis of MM [2,3]. Although the prevalence of kappa and lambda light chains [4,5] or any of the other types of heavy chains is not uncommon, an insidious case presenting with multiple spinal fractures and very high beta-2 microglobulin (β2M) levels with classical clinical features warrants a diagnosis of myeloma. In most phenotypes of MM, plasma cells proliferate with a neoplastic propensity, producing a monoclonal Ig [6]. This plasma cell type is pathognomonic for bone metastasis, causing widespread skeletal damage, osteoporosis, osteopenia, and compression spinal collapse or fracture.

In addition to monoclonal Ig aberration, another important factor in MM pathobiology is increased β2M levels [7]. In humans without any known genetic variation, β2M is present unequivocally in almost all cells and fluids such as the serum, urine, and cerebrospinal fluid (CSF). There are at least two different ways by which β2M is mechanistically involved in the pathogenesis of MM. It is also related to frailty concerning general and skeletal health in individuals over the age of 60 [8].

Structurally, β2M has seven β‑strands organized into two β‑sheets linked by a single disulfide bridge, presenting a classical β‑sandwich resembling Ig. β2M has a distinctive molecular structure, constant‑1 Ig superfamily domain, and immune molecule complexes such as major histocompatibility complex (MHC) class I and II [9]. β2M consists of two molecularly unique tryptophan (Trp) residues that play differential and complementary roles in its structure, guiding it towards the spontaneous and suicidal aggregation into amyloid fibrils [10,11]. Along with interleukin-1β (IL-1β), it shows involvement in bone-related conditions, promoting a cell-mediated calcium efflux causing osteoclast stimulation and osteoblast-osteoclast disbalance favoring lytic destruction and bony metastasis [12]. In this case, the diagnosis became complex and led to a clinical dilemma when a 64-year-old male patient initially presented with clinical, biochemical, radiological, and histopathological features consistent with MM but rapidly recovered and contrasted the previous findings within five months. Informed consent was taken from the patient before preparing this manuscript.

## Case presentation

A 64-year-old, diabetic, non-smoker male patient presented to the emergency department with an acute episode of severe lower back pain, inability to walk, and being completely bedridden. He also complained of difficulty in urination for three days. The patient had generalized swelling with pitting edema in both lower limbs. He was a known patient of type two diabetes mellitus for more than 25 years and was on oral hypoglycemic drugs and insulin. There was no history of trauma or similar episodes of illness, and there was no significant family history. This case discussion is based upon two rounds of workup conducted between March and August 2019. The discussion encompasses the chronological events related to investigations, differential and provisional diagnoses, management plans, treatment, recovery, and follow-up.

Laboratory analysis

**Figure 1:**
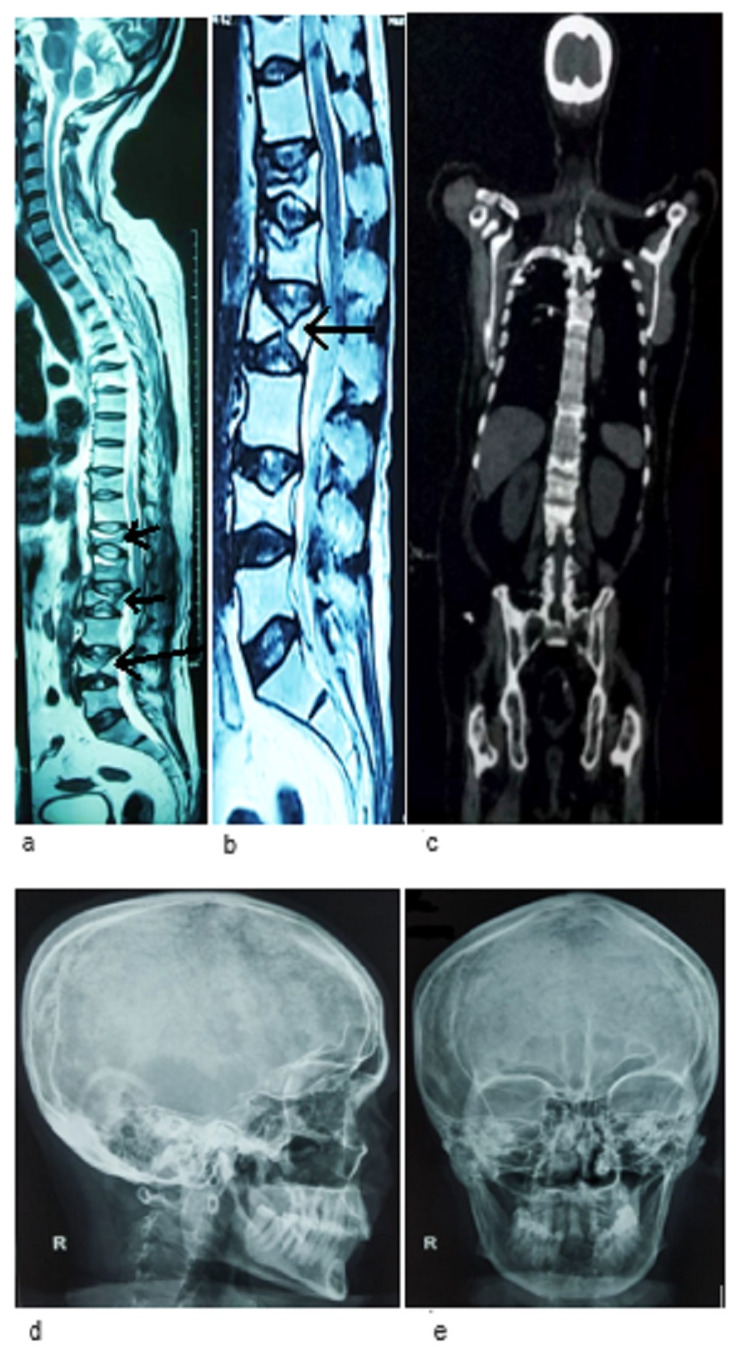
Radiographic findings of the patient. A: MRI of the dorsolumbar spine showing the partial collapse of D5, D6, D10, D12, L1, and L4 vertebrae and irregularity of the endplate with marrow edema. A diffuse disc bulge indenting the anterior thecal sac can be seen at the L1-2, L3-4, L4-5, and L5-S1 levels in association with bilateral neural foramina compression (March 2019). B: MRI of the spine revealing lumbar spondylosis with anterior wedge compression fracture of the L4, L2, L1, D12, D10 vertebral bodies (August 2019). C: PET-CT images showing few inflammatory lymph nodes suspicious for metastasis (March 2019). D, E: Lateral and frontal X-ray images of the skull showing no evidence of lytic or punched-out lesions (March 2019). PET-CT: positron emission tomography-computed tomography; MRI: magnetic resonance imaging

In March 2019, after the first presentation with acute back pain, magnetic resonance imaging (MRI) of the dorsolumbar spine revealed the partial collapse of D5, D6, D10, D12, L1, and L4 vertebrae and the irregularity of endplate with marrow edema. A diffuse disc bulge indenting the anterior thecal sac was noted at the L1-2, L3-4, L4-5, and L5-S1 levels along with bilateral neural foramina compression. Facet joint arthropathy with ligamentum flavum hypertrophy was recorded at multiple levels in the lumbar spine. Screening of the entire spine revealed loss of cervical lordosis with osteophytes and disc desiccation changes at various levels. MRI findings of the spine strongly suggested malignancy (Figures 1A, 1B). The overall spine health was poor, further evidenced by a bone mineral density (BMD) scan with a T-score of -3.5 and a Z-score of -3.1 (Table 1). Whole-body positron emission tomography-computed tomography (PET-CT) images (vertex to mid-thigh) were acquired in the three-dimensional mode. Findings suggested mildly hypermetabolic cervical, axillary, and abdominal lymph nodes, likely inflammatory with partial collapse of multiple vertebrae (Figure 1C). Digital X-rays of the skull, both lateral and frontal view, did not show any abnormality, and there were no signs of punched-out lesions (Figures 1D, 1E).

**Table 1:** Comparative analysis of basic biochemistry and BMD between March 2019 and August 2019. ALP: alkaline phosphatase; ALT: alanine transaminase; AST: aspartate transaminase; BMD: bone mineral density; CRP: C-reactive protein; DEXA: dual-energy X-ray absorptiometry; GGT: gamma-glutamyl transferase; PP: postprandial; PSA: prostate-specific antigen; PTH: parathyroid hormone; RBC: red blood cell; TPO: thyroid peroxidase; TSH: thyroid-stimulating hormone; WBC: white blood cell

Test	March 2019	August 2019	Reference range
Basic biochemistry			
Serum glucose (PP)	246	136	70–140 mg/dL
Serum sodium	141	132.4	136–145 mEq/L
Serum potassium	3.4	4.52	3.5–5.0 mEq/L
Serum chloride	105	99.3	101–109 mEq/L
Serum urea	15	18	17–43 mg/dL
Serum creatinine	0.6	0.58	0.67–1.17 mg/dL
Serum uric acid	3.5	4.55	3.5–7.2 mg/dL
Serum total bilirubin	0.8	0.9	0.3–1.2 mg/dL
Bilirubin conjugated (direct)	0.3	0.2	0.0–0.2 mg/dL
Serum ALT	15	21	10–49 U/L (37°C)
Serum AST	50	29	<34 U/L (37°C)
Serum GGT	13	19	<73 U/L (37°C)
Serum ALP	150	186	30–120 U/L (37°C)
Serum total protein	5.7	6.72	6.4–8.1 g/dL
Serum albumin	2.3	3.6	3.2–4.6 g/dL
Serum calcium (total)	7.8	9.49	8.8–10.2 mg/dL
Serum magnesium	1.6	1.8	1.5–2.5 mg/dL
Serum phosphorous (inorganic)	3.9	4.0	2.5–4.5 mg/dL
Vitamin D (25-OH) (total)	37.52	41.0	>30 ng/mL
Serum CRP	102.8	6.10	<6.00 mg/L
Serum PSA	0.07	0.047	0.07–0.25 ng/mL
Serum TSH	4.93	5.64	0.550–4.780 uIU/mL
Serum PTH (intact)	7.82	19.30	18.50–88.00 pg/mL
Serum anti-TPO	30.1	31.20	<60.00 U/mL
Routine urine analysis			
Proteins	Nil	Nil	Nil
Glucose	Nil	Nil	Nil
Ketones	Nil	Nil	Nil
Bilirubin	Nil	Nil	Nil
Urobilinogen	Normal	Normal	Normal
Leukocyte esterase	Positive	Negative	Negative
Nitrite	Negative	Negative	Negative
Urine microscopy analysis			
RBC	Negative	Negative	Negative
Pus cells	20–25 WBCs/HPF	18–20 WBCs/HPF	0–5 WBCs/HPF
Epithelial cells	Few	Few	Few
Casts	Few	Nil/LPF	Nil/LPF
Crystals	Nil	Nil	Nil
Gross hematuria	Nil	Nil	Nil
Bone density scan (DEXA)			
BMD, L1-4	0.804	0.824	g/cm^2^
T-score, L1-4	-3.5	-1.9	≥-1.0
Z-score, L1-4	-3.1	-0.8	≥-2.0

**Figure 2:**
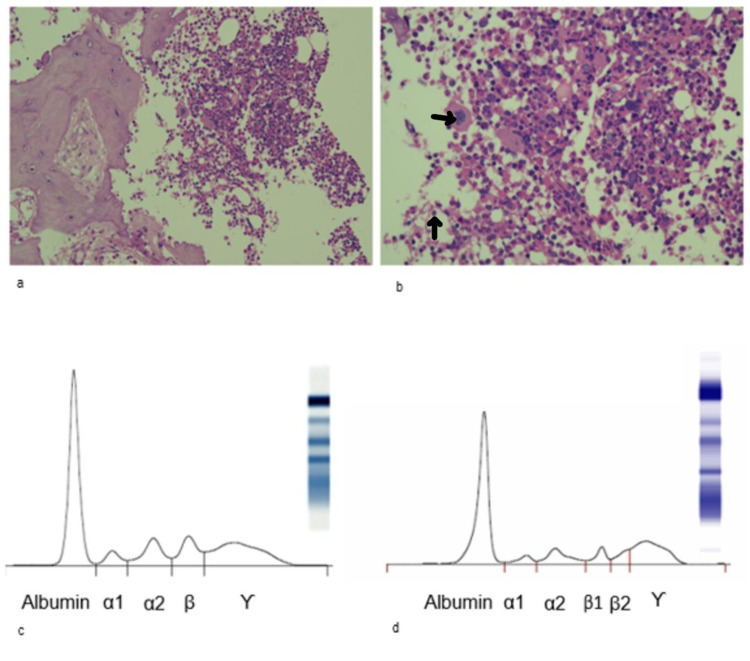
Serum gel electrophoresis pattern and histopathological microplates. A, B (March 2019): images showing marrow spaces of hematopoietic tissue. Hematopoietic cells can be seen in different lineages. There is no evidence of granuloma and perinuclear halo. Atypical or malignant cells are also not seen. C (March 2019) and D (August 2019): serum protein electrophoresis pattern without any anomalies. M-spike is also absent.

Histopathological examination of the specimen obtained from the D10 vertebrae revealed a core of cancellous bone tissue showing trabeculae of the lamellar bone surrounding cellular marrow spaces as well as islands of hematopoietic tissue, mature adipose tissue, and a slight increase in plasma cells. Hematopoietic cells showed all three lineages. There was no evidence of granuloma, atypical, or malignant cells in multiple serial sections; however, this was not entirely conclusive against malignant signatures (Figure 2, Panels A, B). Basic biochemistry, routine, and microscopic urine examination were performed. Postprandial blood glucose and C-reactive protein (CRP) were elevated. Moreover, urine microscopy revealed an increased white blood cell (WBC) count (Table 1).

Standard testing protocols were performed including serum protein electrophoresis, immunotyping, free kappa and lambda light chains (serum and urine) [13]. Serum β2M level was markedly elevated along with elevation of light chains, both in serum and urine (Table 2).

**Table 2:** Comparative analysis of CBC, DLC, ESR, and protein and urine electrophoresis between March 2019 and August 2019. CBC: complete blood count; ESR: erythrocyte sedimentation rate; Ig: immunoglobulin; INR: international normalized ratio; M: myeloma spike; MCH: mean corpuscular hemoglobin; MCHC: mean corpuscular hemoglobin concentration; MCV: mean corpuscular volume; WBC: white blood cell; RBC: red blood cell; RDW: red cell distribution width; PT: prothrombin time

Test	March 2019	August 2019	Reference range
CBC with ESR			
Hemoglobin	11.2	12.9	13.0–17.0 g/dL
Hematocrit	34.1	38.2	40.0–50.0%
WBC count	7.82	6.8	4.0–10.0 10^3^/mm^3^
RBC count	3.97	4.3	4.4–5.5 million/UL
MCV	86.0	86.5	83.0–101.0 FL
MCH	28.3	28	27.0–32.0 PG
MCHC	32.9	33.1	31.5–35 g/dL
RDW	15.6	13.5	11.5–14.5%
Platelet count	273	310	150–410 10^3^/mm^3^
ESR	30	28	<14 mm/first hour
Differential count			
Neutrophils	66.8	60.8	40–80%
Lymphocytes	17.5	16	20–40%
Monocytes	5.8	3.7	2–10%
Eosinophils	9.6	4.9	1–6%
Basophils	0.3	0.3	0–1%
Absolute leukocyte count			
Neutrophils	5.23	6.1	2.0–7.0 × 10^3^/mm^3^
Lymphocytes	1.37	1.5	1.0–3.0 × 10^3^/mm^3^
Monocytes	0.45	0.5	0.2–1.0 × 10^3^/mm^3^
Eosinophils	0.75	0.46	0.02–0.5 × 10^3^/mm^3^
Basophils	0.02	0.1	0–0.1 × 10^3^/mm^3^
Clotting assay			
PT	13.6	13	11–13 seconds
Mean normal PT	12	10	
INR	1.1	1.1	
Protein electrophoresis			
Total protein	6.0	7.2	6.40–8.10 g/dL
Albumin (A)	2.71	3.74	3.60–5.40 g/dL
Alpha 1 globulin	0.31	0.30	0.20–0.40 g/dL
Alpha 2 globulin	0.72	0.75	0.50–1.00 g/dL
Beta 1 globulin	0.70	0.42	0.50–1.10 g/dL
Beta 2 globulin	-	0.38	0.30–0.60 g/dL
Gamma (G) globulin	1.57	1.61	0.70–1.50 g/dL
A:G	0.82	1.08	0.90–2.00 g/dL
M spike	Not seen	Not seen	Not seen
Beta-2 microglobulin	5,450	900	700–1,800 ng/mL
Immunoglobulin profile			
IgG	16.7	13.2	7.0–16.0 g/L
IgA	2.62	2.89	0.7–4.0 g/L
IgM	0.56	0.5	0.4–2.3 g/L
Light chain (serum analysis)			
Free kappa (light chain)	57.3	21.2	6.7–22.4 mg/L
Free lambda (light chain)	56.4	18.4	8.3–27.0 mg/L
Free kappa/lambda ratio	1.02	1.15	0.31–1.56
Immunofixation panel (urine)			
Free kappa (light chain)	1,390	21.3	1.35–24.19 mg/L
Free lambda (light chain)	82.40	6.5	0.24–6.66 mg/L
Free kappa/lambda ratio	16.87	3.27	2.04–10.37
Protein electrophoresis (24-hour urine)			
Total proteins	1,536.25	128	28–141 mg/24 hours
Albumin	88.5	-	-
Alpha 1	11.5	-	-
Alpha 2	-	-	-
Beta	-	-	-
Gamma	-	-	-
M spike	Nil	Nil	Nil
Gel electrophoresis (24-hour urine)			
Electrophoretic zone	Absent	Absent	Absent
IgG + IgM + IgA	Absent	Absent	Absent
Free and bound kappa	Absent	Absent	Absent
Free and bound lambda	Absent	Absent	Absent
Free kappa	Absent	Absent	Absent
Free lambda	Absent	Absent	Absent
Bence Jones protein (urine)	Absent	Absent	Absent

Serum protein gel electrophoresis did not show any abnormal band or M-spike (Figure 2, Panel C). After the first round of workup on the patient in March 2019, he was followed up in August 2019. Basic biochemistry, urine examination, complete blood count (CBC), and BMD were performed (Table 1). MRI of the dorsolumbar spine, serum protein electrophoresis, gel fixation (Figure 2, Panel D), immunofixation, light chain estimation (serum and urine), immunotyping, and β2M estimation were repeated (Table 2).

Differential diagnosis and treatment

Based upon the clinical features and laboratory findings, we considered multiple myeloma, tuberculosis of the spine, metastatic prostate carcinoma, lymphoplasmacytoid lymphoma, disuse osteoporosis, and isolated β2M-associated osteoporotic compression fracture. The patient was given symptomatic treatment when he first presented in March 2019. Initially, baseline correction was done for electrolytes, blood glucose, and other constitutional symptoms. The patient was on disease-modifying drugs such as ibandronate, calcium, and vitamin D supplements. Physiotherapy for flexibility, spinal range of motion, and strengthening exercises were advised. The patient was followed up in August 2019. On follow-up, his physical condition had improved, and he could walk and perform daily activities without support. Moreover, his pain was entirely resolved.

## Discussion

This is a typical case that addresses the dilemma raised in scenarios of unclear diagnosis and tentative treatment plans. When this patient first presented in March 2019, his physical and clinical findings were suggestive of a diagnosis of MM. Numerous spinal fractures, poor bone density, clinical symptoms, a very high level of β2M, and unclear histopathological findings led us to consider malignancy and plan appropriate treatment. Even when we weighed the second option of excluding myeloma, the vertebrae health was very fragile; hence, we planned for bone cementing between L1-4 to preserve the long-term mobility and the general health of the patient. However, the absence of malignant cells in core needle vertebral biopsy, no relevant findings on PET-CT scan, no lytic lesion on the skull, lack of M-band in electrophoresis, and no malignancy-related anomalies on immunofixation and immunotyping compelled us to think otherwise. We ruled out prostate cancer based on prostate-specific antigen levels along with Gleason’s score of less <7 on a digital rectal examination [14]. Tuberculosis of the spine was ruled out based on the clinical, biochemical, microbiological, and radiological findings. Secondary metastasis and lymphoplasmacytoid lymphoma were ruled out because of the absence of any metastasis and blood findings. Disuse osteoporosis is a subjective diagnosis and can exist along with any of the differential diagnoses mentioned earlier. After five months, when the patient presented for a follow-up, his recovery was remarkable. On follow-up, he walked without support to the outpatient department. MRI of the spine showed improvement, BMD showed improved density, and CRP along with most of his important biochemistry markers had reached the baseline. Most remarkably, the β2M, serum and urinary proteins, and kappa and lambda light-chain (serum and urine) levels were within the normal range (Tables 1, 2).

The differential diagnoses were ruled out based on the above observations and findings, and a final diagnosis of multiple spinal osteoporotic fractures was made.

Learning points

According to the doctrine of Occam, multiple entities should not be considered without necessity. In this context, it is always better to consider a single working diagnosis and follow a single treatment plan, even for complex diseases [15]. However, at the same time, confirming a diagnosis can be challenging. Other than MM, the differential diagnoses include secondary metastasis from an occult primary, lymphoma, and infections such as tuberculosis [16]. We constantly reviewed the patient, with the view to make a specific diagnosis, we were concerned because of the uncertain diagnosis. Although osteoporotic fracture, MM, and elevated β2M are not uncommon, together they can raise clinical confusion in a frail patient.

## Conclusions

Our patient thought that he might not walk again in his life. We weighed all the options for interventions. Additionally, we assessed his condition with careful and critical analysis, performed all the necessary investigations, excluded MM, and opted for non-surgical conservative management. The treatment plan led to the remarkable recovery of the patient.
